# A Functional Polymorphism in Accessible Chromatin Region Confers Risk of Non-Small Cell Lung Cancer in Chinese Population

**DOI:** 10.3389/fonc.2021.698993

**Published:** 2021-09-06

**Authors:** Jieyi Long, Tingting Long, Ying Li, Peihong Yuan, Ke Liu, Jiaoyuan Li, Liming Cheng

**Affiliations:** Department of Laboratory Medicine, Tongji Hospital, Tongji Medical College, Huazhong University of Science and Technology, Wuhan, China

**Keywords:** chromatin accessibility, single-nucleotide polymorphism, non-small cell lung cancer, susceptibility, heterochromatin protein 1 gamma

## Abstract

**Background:**

The disease-associated non-coding variants identified by genome-wide association studies (GWASs) were enriched in open chromatin regions (OCRs) and implicated in gene regulation. Genetic variants in OCRs thus may exert regulatory functions and contribute to non-small cell lung cancer (NSCLC) susceptibility.

**Objective:**

To fine map potential functional variants in GWAS loci that contribute to NSCLC predisposition using chromatin accessibility and histone modification data and explore their functions by population study and biochemical experimental analyses.

**Methods:**

We mapped the chromatin accessible regions of lung tissues using data of assay for transposase-accessible chromatin using sequencing (ATAC-seq) in The Cancer Genome Atlas (TCGA) and prioritized potential regulatory variants within lung cancer GWAS loci by aligning with histone signatures using data of chromatin immunoprecipitation assays followed by sequencing (ChIP-seq) in the Encyclopedia of DNA Elements (ENCODE). A two-stage case–control study with 1,830 cases and 2,001 controls was conducted to explore the associations between candidate variants and NSCLC risk in Chinese population. Bioinformatic annotations and biochemical experiments were performed to further reveal the potential functions of significant variants.

**Results:**

Sixteen potential functional single-nucleotide polymorphisms (SNPs) were selected as candidates from bioinformatics analyses. Three variants out of the 16 candidate SNPs survived after genotyping in stage 1 case–control study, and only the results of SNP rs13064999 were successfully validated in the analyses of stage 2 case–control study. In combined analyses, rs13064999 was significantly associated with NSCLC risk [additive model; odds ratio (OR) = 1.17; 95%CI, 1.07–1.29; *p* = 0.001]. Functional annotations indicated its potential enhancer bioactivity, and dual-luciferase reporter assays revealed a significant increase in luciferase activity for the reconstructed plasmid with rs13064999 A allele, when compared to the one with wild-type G allele (*p_A549_* < 0.001, *p_SK-MES-1_* = 0.004). Further electrophoretic mobility shift assays (EMSA) and super-shift assays confirmed a stronger affinity of HP1γ for the binding motif containing SNP rs13064999 A allele.

**Conclusion:**

These findings suggested that the functional variant rs13064999, identified by the integration of ATAC-seq and ChIP-seq data, contributes to the susceptibility of NSCLC by affecting HP1γ binding, while the exact biological mechanism awaits further exploration.

## Introduction

Lung cancer is a complex disorder resulting from multifactors. Despite environmental risk factors such as cigarette smoking, accumulating evidence have provided a crucial role of genetic components to the etiology of lung cancer (1). As an effective approach to explore genetic architecture of complex traits, genome-wide association studies (GWASs) so far have identified 45 susceptibility loci involved in lung cancer (2). However, the causal variants and underlying mechanisms are largely obscure. Indeed, the vast majority of GWAS signals are non-coding variants, which have been demonstrated to change gene expression by modulating cis-regulatory machineries through multiple mechanisms (3). Thus, it is an imperative task and a big challenge to prioritize causal single-nucleotide polymorphism (SNPs) with regulatory functions in post-GWAS era.

Open chromatin regions (OCRs), which cover 90% of regions bound by transcriptional factors (TFs), contain various cis-regulatory elements (CREs), such as enhancers, promoters, and insulators (4, 5). Due to this property, OCR was regarded as a hallmark of regulatory elements. Interestingly, it has been suggested that SNPs relevant to complex diseases were enriched in these regions (6). For example, studies in human islet cells have showed that SNPs associated with type 2 diabetes (T2D) reside in OCRs and enhancers (7–9). Similarly, schizophrenia risk genetic variants were also found to gather at OCRs of human glutamatergic neurons (10). Therefore, it seems that open chromatin serves as a putative region to prioritize functional polymorphisms.

With a simple procedure and a low number of cells input, assay for transposase-accessible chromatin with high-throughput sequencing (ATAC-seq) has emerged as the most popular approach to capture open chromatin (11). More importantly, a higher sensitivity and signal-to-noise ratio enable ATAC-seq to map open chromatin more precisely than other techniques such as DNase I hypersensitive sites sequencing (DNase-Seq) and formaldehyde-assisted isolation of regulatory elements sequencing (FAIRE-seq). By ATAC-seq profiling, a large number of disease-relevant variants in OCRs have been identified (12–14). However, as ATAC-seq only unravels latent regulatory regions, it is usually necessary to integrate other functional genomic data with ATAC-seq to narrow down a list of regulatory SNPs. Through the integration of ATAC-seq and H3K27ac and H3K4me2 chromatin immunoprecipitation sequencing (ChIP-seq), coronary artery disease- or ischemic stroke-associated SNP rs17114036 was predicted to fall within an enhancer element. Subsequent functional experiments suggested that rs17114036 allele C increased enhancer activity and upregulated *PLPP3* by changing the KLF2 binding sites (15). In addition, a combination of ATAC-seq, ChIP-seq, and Hi-C data has been used to map target genes of islet enhancers, in which a T2D variant rs10428126 was found to confer the reduction in enhancer activity and *IGF2BP2* expression (16). Thus, mapping regulatory elements in open chromatin by ATAC-seq and other functional features may facilitate identification of causative SNPs in common disease predisposition.

In this study, to identify functional polymorphisms that contribute to non-small cell lung cancer (NSCLC) susceptibility, we performed an integrative analysis of ATAC-seq and ChIP-seq data to select candidate SNPs within GWAS loci. Next, a two-stage case–control study was conducted to evaluate and validate the associations between candidate SNPs and NSCLC susceptibility. Afterward, biological experiments were introduced to further clarify the potential functions of the significant variant.

## Materials and Methods

### Screening of Candidate Variants

Firstly, we downloaded NSCLC-associated tag SNPs identified by GWASs in East Asians from US National Human Genome Research Institute Catalog of Published GWAS (http://www.ebi.ac.uk/gwas/) up to March 30, 2019. Given the potential transcriptional regulatory function of subthreshold GWAS signals as reported (17), both SNPs with genome-wide significance (*p* < 5 × 10^−8^) and subthreshold significance (5 × 10^−8^ < *p* < 10^-5^) were retrieved. Next, the variants in linkage disequilibrium (LD) with the tag SNPs were detected by HaploView software (setting r^2^ > 0.2). Then, we mapped the variants within chromatin accessible regions by ATAC-seq data of lung tissue from The Cancer Genome Atlas (TCGA) database (https://portal.gdc.cancer.gov/). To further narrow down the extent of potential functional polymorphisms, we additionally integrated with histone signature H3K27ac by ChIP-seq data of lung cancer cell line A549 from Encyclopedia of DNA Elements (ENCODE) project (https://www.encodeproject.org/).

### Study Participants

We performed a two-stage case–control study to evaluate the associations between candidate SNPs and NSCLC susceptibility. A total of 348 NSCLC patients and 479 cancer-free controls were enrolled for discovering the promising variants in stage 1 case–control study. In stage 2, we newly recruited 1,482 NSCLC cases and 1,522 cancer-free controls to validate the associations. All participants were consecutively enrolled from January 2014 to January 2018 in the Tongji Hospital of Huazhong University of Science and Technology (HUST). The cases were histopathologically or cytologically diagnosed by pathologists, without previous surgery, chemotherapy, or radiotherapy prior to the collection of blood samples. During the same time with the cases enrolled, controls were randomly selected by a healthy screening in the same hospital and matched to cases by gender and age ( ± 5 years). The exclusion criteria for controls included any forms of cancers, precancerous lesions, or serious illness. Basic demographic characteristics of the participants including age, sex, and smoking status were obtained from medical records or interviews. Individuals were defined as non-smokers if they have never smoked or smoked <1 cigarette per day for <1 year until the date of interview for controls or cancer diagnosis for cases; otherwise, they were classified as smokers. This study was approved by the Institutional Review Committee of the Tongji Hospital, Tongji Medical College, HUST.

### Genotyping

Two milliliter peripheral blood sample was collected from each participant. We used the RelaxGene Blood System DP319-02 (Tiangen, Beijing, China) to extract genomic DNA. The quality of DNA was assessed by NanoDrop 2000 spectrophotometer (Thermo Fisher Scientific, Waltham, MA, USA). In stage 1, candidate SNPs were genotyped using the Sequenom MassARRAY system (SEQUENOM, San Diego, CA, USA). In stage 2, promising SNPs were genotyped by Taqman SNP Genotyping Assay on Roche LightCycler480 (Roche, Basel, Switzerland). Quality control was conducted by 5% duplicates and negative controls in each plate of samples.

### Cell Lines and Cell Culture

Human lung cancer cell lines A549 and SK-MES-1 were provided by Stem Cell Bank, Chinese Academy of Sciences (Shanghai, China). Before being used in experiments, cell lines were tested for the absence of mycoplasma contamination. The A549 cells and SK-MES-1 cells were grown in Roswell Park Memorial Institute (RPMI) 1640 (Gibco, Carlsbad, CA, USA) and Dulbecco’s modified Eagle’s medium (Gibco), respectively. Both cell lines were supplemented with 10% fetal bovine serum (FBS) and maintained at 37°C in a humidified atmosphere with 5% CO_2_. The cells used in experiments have never been passaged longer than 1 month since thawing.

### Plasmid Reconstruction

The wild-type sequence of 500 bp within both sides of the SNP rs13064999 (G allele) was downloaded from the National Center for Biotechnology Information (NCBI) database and commercially synthesized by Genewiz (Suzhou, China). Synthesized products were subsequently subcloned into the *Kpn*I and *Xho*I sites of the pGL3-Promoter vector. The mutant sequence corresponding to genetic variant rs13064999 (A allele) was generated by site-specific mutagenesis and then cloned into the same position of pGL3-Promoter vector.

### Dual-Luciferase Assays

Cells were seeded in 96-well flat-bottomed plates and grown for 24 h to reach approximately 80% confluence. Negative control pGL3-Promoter, reconstructed plasmid containing rs13064999 wild type or mutant type were respectively cotransfected with pRL-SV40 vector using Lipofectamine 3000 (Invitrogen, Waltham, MA, USA). Twenty-four hours after transfection, luciferase activity was measured using the Dual-Luciferase Reporter Assay System (Promega, Madison, WI, USA). Renilla luciferase and firefly luciferase activities were detected, and relative luciferase activity was calculated. Three independent experiments were performed, and triplicate wells were transfected in each experiment.

### Electrophoretic Mobility Shift Assays

Complementary DNA oligonucleotides used for electrophoretic mobility shift assays (EMSA) were synthesized (Takara, Beijing, China) and labeled with or without biotin at the 3′ end. Nuclear proteins of A549 and SK-MES-1 cells were prepared according to the protocol for the Nuclear and Cytoplasmic Protein Extraction Kit (Beyotime, Shanghai, China). The protein concentrations of the nuclear extracts were determined by bicinchoninic acid (BCA) protein assay kit (Beyotime) and stored in −80°C until use. Labeled probes were incubated with nuclear extracts on ice for 15 min. The competition assays were performed by adding 40- or 400-fold excess of unlabeled probe against the labeled probes. For super-shift assays, rabbit monoclonal antiheterochromatin protein 1 gamma (HP1γ) immunoglobulin G (IgG) antibody (Abcam ab217999, Cambridge, UK) or rabbit monoclonal IgG isotype control (Abcam ab172730, Cambridge, UK) was incubated with nuclear extracts before adding labeled DNA probes. The DNA–protein complexes were separated on 6% native polyacrylamide gel and transferred to nylon membrane. Following the UV cross-link, biotinylated oligonucleotides were detected by chemiluminescence with the SuperSignal™ West Femto Substrate Trial Kit (Thermo Fisher Scientific, Waltham, MA, USA).

### Statistical Analysis

The Hardy–Weinberg equilibrium (HWE) for genotypes in controls was assessed by a goodness-of-fit *χ* ^2^ test. Pearson’s *χ* ^2^ test was used to examine the differences in distributions of genotypes between cases and controls. Unconditional multivariate logistic regression adjusted for age, sex, and smoking status was adopted to evaluate the association between each SNP and NSCLC risk. The genetic models including homozygous, heterozygous, dominant, recessive, and additive models were applied for the association analyses. For reporter gene assay, comparisons between wild type and variant allele of rs13064999 were evaluated by Student’s *t*-test. The statistical results and the corresponding images were acquired using GraphPad Prism v7.04. The association analyses were performed with SPSS 25.0 and Plink v1.90. A two-tailed *p* < 0.05 was used as the criterion of statistical significance. Of note, owing to the limited sample size in stage 1, *p* < 0.1 was considered as the threshold for selecting promising SNPs.

## Results

### Selection of Candidate SNPs

The process of candidate SNP selection is presented in **Supplementary Figure S1**. In total, 28 tag SNPs were retrieved from GWAS catalog, including 21 significant variants and 7 subthreshold ones. To avoid missing potential causal variants linked with the significant variant, we subsequently obtained 4,069 LD variants by Haploview. The position information of ATAC-seq data from 22 lung cancer tissues (Project: TCGA-LUAD) were download from TCGA database, and a total of 256,453 segments on autosomes were captured by sequencing. H3K27ac data by ChIP-seq of lung cancer cell line A549 with no treatment (Experiment ID: ENCSR778NQS) were retrieved from ENCODE project. The experiment identified 63,241 signals for H3K27ac histone modifications in the whole genome. By aligning the positions of the 4,069 SNPs in GWAS loci with ATAC-seq locations, we predicted 232 NSCLC-associated variants that reside within open chromatin. Further alignment with the H3K27ac locations narrowed down the candidates to 16 variants with potential regulatory functions. **Supplementary Table S1** shows the basic information of 16 candidate SNPs.

### Characteristics of Study Subjects

Demographic characteristics of the study participants are summarized in **Table 1**. Briefly, a total of 1,830 NSCLC patients and 2,001 healthy controls were enrolled for genotyping in this study. No significant difference was found between cases and controls for age (55.3 ± 8.6 years for cases, 55.0 ± 8.5 years for controls, *p* = 0.281) and sex (63.6% male in cases, 63.8% male in controls, *p* = 0.889). Compared to healthy controls, the proportion of smokers was marginally or significantly higher in cases (*p*_stage1_ = 0.052; *p*_stage2_ < 0.001; *p*_combined population_ < 0.001). Of all cases, 65.1% were adenocarcinoma, 26.2% were squamous cell carcinoma, and others were adenosquamous carcinoma, large cell lung cancer, and a small part of NSCLC cases who could not be classified.

**Table 1:** Characteristics of subjects in the two-stage case–control study.

	Stage 1				Stage 2				Combined population			
	Case	Control	χ^2^	*p*	Case	Control	χ^2^	*p*	Case	Control	χ^2^	*p*
Total	348	479			1,482	1,522			1,830	2,001		
Age (mean ± SD)	56.3 ± 7.8	55.2 ± 9.7		0.083	55.1 ± 8.7	54.9 ± 8.1		0.652	55.3 ± 8.6	55.0 ± 8.5		0.281
Sex, n (%)												
Male	196 (56.3)	270 (56.4)	0.0	0.990	967 (65.2)	1,006 (66.1)	0.24	0.625	1,163 (63.6)	1,276 (63.8)	0.02	0.889
Female	152 (43.7)	209 (43.6)			515 (34.8)	516 (33.9)			667 (36.4)	725 (36.2)		
Smoking status, n (%)												
Smoker	145 (41.7)	160 (33.4)	5.91	0.052	736 (49.7)	628 (41.3)	23.80	<0.001	881 (48.1)	788 (39.4)	30.96	<0.001
Non-smoker	198 (56.9)	311 (64.9)			734 (49.5)	887 (58.3)			932 (50.9)	1,198 (59.9)		
Unknown	5 (1.4)	8 (1.7)			12 (0.8)	7 (0.5)			17 (0.9)	15 (0.7)		
Histology, n (%)												
Adenocarcinoma	256 (73.6)				936 (63.2)				1,192 (65.1)			
Squamous cell carcinoma	76 (21.8)				415 (28.0)				491 (26.8)			
Other	16 (4.6)				131 (8.9)				147 (8.0)			

### Association Between Individual Candidate Variant and NSCLC Susceptibility

In stage 1, the variant rs34417254 was rejected for genotyping due to design failure in the reaction system. Then, rs62085661 with a high LD with rs34417254 (r^2^ = 1) in Asian population was used to be a substitute. Owing to the deviation from the HWE in controls (*p* < 0.05), the variant rs2290368 was excluded. Simultaneously, two SNPs (rs7502307 and rs9908003) were removed for the genotyping call rates <90%. The associations between the rest 13 SNPs and NSCLC risk are shown in **Table 2**. Among the 13 variants, 3 SNPs were reached our threshold (*p* < 0.1) for promising associations with NSCLC susceptibility under the additive model, including rs13064999 [odds ratio (OR) = 1.21; 95%CI, 0.98–1.48; *p* = 0.072], rs12752 (OR = 0.83; 95%CI, 0.67–1.03; *p* = 0.097] and rs62085661 (OR = 0.82; 95%CI, 0.66–1.02; *p* = 0.072).

**Table 2:** Results of association analyses between individual SNPs and NSCLC risk in stage 1.

rs ID	*p*_HWE_	MAF		*χ* ^2^	*p* ^a^	OR (95%CI)^b^	*p* ^b^
		Case	Control				
rs13064999	0.922	0.41	0.37	3.19	0.074	1.21 (0.98–1.48)	0.072
rs62290287	0.516	0.45	0.45	0.02	0.881	0.98 (0.80–1.19)	0.814
rs71317943	0.729	0.26	0.27	0.12	0.727	0.98 (0.77–1.23)	0.886
rs4946258	0.515	0.42	0.45	1.50	0.221	0.89 (0.72–1.09)	0.244
rs11169971	0.542	0.35	0.34	0.04	0.835	1.02 (0.83–1.25)	0.880
rs8079078	0.072	0.20	0.20	0.05	0.828	1.02 (0.78–1.33)	0.902
rs34122828	0.892	0.18	0.21	2.40	0.122	0.82 (0.64–1.05)	0.119
rs151235307	0.842	0.15	0.13	0.81	0.369	1.13 (0.85**–**1.51)	0.384
rs12752	0.523	0.28	0.31	2.51	0.113	0.83 (0.67**–**1.03)	0.097
rs62085661	0.396	0.28	0.32	2.79	0.095	0.82 (0.66**–**1.02)	0.072
rs12601492	0.925	0.44	0.42	0.44	0.505	1.06 (0.87**–**1.30)	0.541
rs9906439	0.103	0.24	0.21	1.16	0.282	1.12 (0.88**–**1.41)	0.351
rs3794742	0.170	0.47	0.49	0.65	0.421	0.91 (0.75**–**1.11)	0.345

95%CI, 95% confidence interval; OR, odds ratio.

a Pearson’s χ^2^ test for distributions of genotypes.

b Logistic analysis in an additive model and adjusted for age, sex, and smoking status.

Given the small sample size in stage 1, the associations between above three variants and NSCLC were further validated in replication stage. As shown in **Table 3**, we found that only SNP rs13064999 was significantly associated with the risk of NSCLC. Under the homozygous, recessive, and additive models, the ORs and 95%CIs were 1.40 (95%CI, 1.12–1.76; *p* = 0.003), 1.34 (95%CI, 1.09–1.65; *p* = 0.006), and 1.16 (95%CI, 1.04–1.29; *p* = 0.006), respectively. The associations remained significant when combining the samples of the two stages together (homozygous model, OR = 1.42; 95%CI, 1.16–1.73; *p* = 0.001; dominant model, OR = 1.17; 95%CI, 1.03–1.34; *p* = 0.020; recessive model, OR = 1.34; 95%CI, 1.12–1.61; *p* = 0.002; additive model, OR = 1.17; 95%CI, 1.07–1.29, *p* = 0.001).

**Table 3:** Association analyses between three SNPs and NSCLC risk in stage 2 and combined population.

	Stage 2				Combined population			
	Case	Control	OR (95%CI)^a^	*p*	Case	Control	OR (95%CI)^a^	*p*
rs13064999								
GG	513	583	1.00		632	770	1.00	
GA	668	707	1.09 (0.97–1.28)	0.313	839	931	1.11 (0.96–1.27)	0.168
AA	245	197	1.40 (1.12–1.76)	0.003	303	261	1.42 (1.16–1.73)	0.001
Dominant model			1.16 (0.99–1.35)	0.062			1.17 (1.03–1.34)	0.020
Recessive model			1.34 (1.09–1.65)	0.006			1.34 (1.12–1.61)	0.002
Additive model			1.16 (1.04–1.29)	0.006			1.17 (1.07–1.29)	0.001
rs12752								
TT	136	137	1.00		166	180	1.00	
TC	616	608	1.00 (0.77–1.30)	0.989	748	819	0.99 (0.78–1.26)	0.951
CC	712	714	0.99 (0.76–1.28)	0.916	898	936	1.04 (0.82–1.31)	0.750
Dominant model			1.01 (0.87–1.17)	0.867			0.96 (0.84–1.09)	0.507
Recessive model			1.01 (0.78–1.30)	0.947			0.98 (0.79–1.23)	0.882
Additive model			1.01 (0.90–1.13)	0.875			0.97 (0.88–1.07)	0.565
rs62085661								
CC	709	728	1.00		891	946	1.00	
CG	604	634	0.98 (0.84–1.14)	0.766	734	849	0.92 (0.80–1.06)	0.233
GG	130	138	0.98 (0.75–1.27)	0.860	160	181	0.93 (0.74–1.18)	0.565
Dominant model			0.98 (0.84–1.13)	0.753			0.92 (0.81–1.05)	0.224
Recessive model			0.99 (0.77–1.27)	0.921			0.97 (0.77–1.22)	0.791
Additive model			0.98 (0.88–1.10)	0.775			0.95 (0.86–1.05)	0.292

All the ORs and p-values were adjusted for sex, age, and smoking status.

a ORs and 95% CIs calculations were conducted under assumption that variant alleles were risk alleles.

**Table 4** presents the subgroup analysis stratified by histology, smoking status, and sex. Our results supported a robust association between rs13064999 and adenocarcinoma risk (additive model, OR = 1.23; 95%CI, 1.10–1.38; *p* < 0.001), while no significant association was observed with squamous cell carcinoma (additive model, OR = 1.07; 95%CI, 0.91–1.24; *p* = 0.430). When the analyses were performed by smoking status, significant association between rs13064999 and NSCLC was only found in nonsmokers (additive model, OR = 1.22; 95%CI, 1.08–1.39; *p* = 0.002) but not in smokers (additive model, OR = 1.10; 95%CI, 0.96–1.27; *p* = 0.176). In the stratified analyses according to sex, we observed a sex difference for rs13064999 and NSCLC risk, with a stronger association among female subjects (additive model, OR = 1.27; 95%CI, 1.08–1.48; *p*= 0.003), as compared to male subjects (additive model, OR = 1.12; 95%CI, 0.99–1.26; *p* = 0.070).

**Table 4:** Subgroup analysis of SNP rs13064999 and NSCLC risk stratified by histology, smoking status, and sex.

	HT *vs*. HW		HV *vs*. HW		Dominant model		Recessive model		Additive model	
	OR (95%CI)^a^	*p*	OR (95%CI)^a^	*p*	OR (95%CI)^a^	*p*	OR (95%CI)^a^	*p*	OR (95%CI)^a^	*p*
Histologic type										
Adenocarcinoma	1.21 (1.03–1.42)	0.024	1.52 (1.22–1.90)	<0.001	1.28 (1.09–1.49)	0.002	1.37 (1.12–1.68)	0.003	1.23 (1.10–1.38)	<0.001
SCC^b^	0.97 (0.76–1.22)	0.762	1.20 (0.87–1.66)	0.268	1.02 (0.82–1.27)	0.884	1.22 (0.91–1.64)	0.180	1.07 (0.91–1.24)	0.430
Smoking status										
Smoker	1.02 (0.83–1.26)	0.854	1.28 (0.94–1.72)	0.113	1.08 (0.88–1.32)	0.480	1.26 (0.96–1.66)	0.099	1.10 (0.96–1.27)	0.176
Non-smoker	1.17 (0.97–1.42)	0.107	1.53 (1.18–2.00)	0.002	1.25 (1.04–1.50)	0.016	1.40 (1.10–1.79)	0.006	1.22 (1.08–1.39)	0.002
Sex										
Male	1.03 (0.86–1.23)	0.751	1.30 (1.02–1.67)	0.037	1.09 (0.92–1.29)	0.318	1.28 (1.02–1.61)	0.032	1.12 (0.99–1.26)	0.070
Female	1.23 (0.97–1.56)	0.084	1.63 (1.17–2.26)	0.004	1.31 (1.05–1.64)	0.016	1.45 (1.07–1.96)	0.016	1.27 (1.08–1.48)	0.003

All the ORs and p-values were adjusted for sex, age, and smoking status.

HW, wild-type homozygote; HT, heterozygote; HV, variant homozygote.

a ORs and 95% CIs calculations were conducted under assumption that variant alleles were risk alleles.

b Squamous cell carcinoma.

### Function Annotation of rs13064999

**Figure 1:**
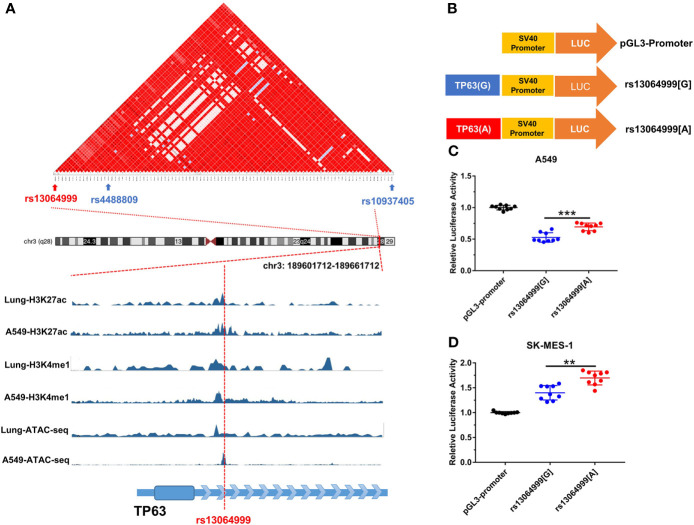
The variant rs13064999 influence gene transcription. **(A)** The bioinformatic analysis of rs13064999. Top, LD map of a total of 141 common SNPs in the 3q28 locus. Bottom, epigenetic tracks for the region surrounding SNP rs13064999 from ENCODE database. **(B)** The diagram of the recombinant plasmid used in luciferase assays. DNA fragments containing rs13064999[G/A] were cloned into pGL3-Promoter vector. **(C, D)** Relative reporter gene activity of the constructs containing rs13064999 G or A allele in A549 cells **(C)** and SK-MES-1 cells **(D)**, respectively. Data were presented as mean ± SD from three independent experiments, each in triplicate. ***p < 0.001, **p < 0.01; *p*-values were calculated by a two-sided Student’s *t*-test.

SNP rs13064999 lies in the intron 1 of the gene *TP63* at 3q28 region, where rs4488809 and rs10937405 were reported to be associated with lung cancer predisposition. Based on 1000 Genomes Project (Phase 3) data in East Asian population, our LD analysis showed that tag SNP rs4488809 and rs13064999 were located in a LD haplotype block. Rs13064999 was in moderate LD with rs4488809 (r^2^ = 0.60, D′ = 0.92) and a weak LD with rs10937405 (r^2^ = 0.22, D′ = 0.87). According to the epigenomic information from ENCODE database, rs13064999 was marked by active enhancer histone modification (H3K27ac and H3K4me1) and located in open chromatin region in the normal lung tissue and lung cancer cell line A549 (**Figure 1A**).

### Transcriptional Activity of rs13064999 Variation

We performed luciferase reporter assays to evaluate whether the variant rs13064999 influences the transcription activity (**Figure 1B**). As shown in **Figures 1C, D**, the constructs containing rs13064999 A allele exhibited higher luciferase activity than that containing the rs13064999 G allele in both cell lines (*p* < 0.001 and *p* = 0.004 for A549 and SK-MES-1 cell lines, respectively).

### Allele-Specific Protein Binding of rs13064999

**Figure 2:**
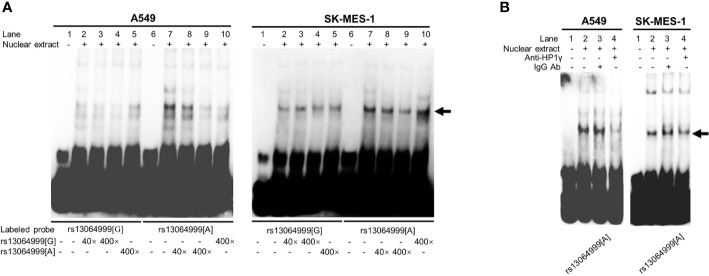
The variant rs13064999 showed an allele-specific binding to HP1γ. **(A)** EMSAs with biotin-labeled probes containing rs13064999-G or A allele in A549 and SK-MES-1 cell line. Lanes 1 and 6, biotin-labeled probe only; lanes 2 and 7, biotin-labeled probe and nuclear extracts; lanes 3–5 and 8–10, biotin-labeled probe, nuclear extracts plus 40× or 400× unlabeled competitor. Arrow indicates the allele-specific bands that interact with cells nuclear extracts. **(B)** Super-shift EMSA assays with the anti-HP1γ antibody in A549 and SK-MES-1 cells. Arrow indicates specific binding complexes to the A allele of rs13064999.

EMSA assays were conducted to investigate whether the binding of transcription factors could be affected by SNP rs13064999. We observed that the rs13064999 A allele was preferentially bound to nuclear extracts compared to the wild G allele in both cell lines. Moreover, the binding signal was gradually attenuated in a dose-dependent manner with the addition of the unlabeled probe containing the rs13064999 A allele (**Figure 2A**). To identify which protein might specifically bind to the motif containing rs13064999 A allele, we firstly conducted a TF binding site prediction analysis using Animal TFDB database (http://bioinfo.life.hust.edu.cn/AnimalTFDB/#!/search). Apart from collecting multiple motif matrix from hTFtarget (http://bioinfo.life.hust.edu.cn/hTFtarget/) and other resources such as HOCOMOCO, JASPAR and TRANSFAC, the database also predicted TF binding motifs based on ChIP-seq data using bioinformatics method (18). As a result, HP1γ was predicted as a candidate factor that specifically binds to the rs13064999 A allele. Notably, several high-confident binding motifs of HP1γ were retrieved through bioinformatics analysis using ChIP-seq data (GSE32465) in the database. Although HP1γ has been considered as a chromatin remodeling regulator, evidence has suggested that HP1γ was also involved in transcriptional initiation, elongation, and termination (19–21). Thus, we further conducted super-shift assays to determine whether HP1γ binds to the rs13064999 A allele. As shown in **Figure 2B**, when compared to IgG isotype control, the addition of antibody against HP1γ attenuated the rs13064999 A binding signal, suggesting an allele-specific affinity of the motif containing rs13064999 A allele binding to HP1γ.

## Discussion

In this study, we attempted to search for potential causal variants in susceptibility loci identified by lung cancer GWASs though integrating ATAC-seq signals and histone markers. Genotyping of the candidate SNPs in a two-stage case–control study revealed that the variant rs13064999 was significantly associated with NSCLC risk. Subsequent functional analyses elucidated its regulatory role in gene transcription by affecting HP1γ binding in an allele-specific manner.

At present, ATAC-seq has been extensively applied to investigate accessible chromatin landscape (22), early embryos development (23), and epigenetic mechanism of tumorigenesis (24). Recent work has proved enormous potential to explore causal SNPs by integrating ATAC-seq and other functional genomic data. For example, with the combination of ATAC-seq and whole genome sequencing (WGS) data, three *TERT* promoter mutations were found to generate ETF motif sites, leading to an increase in *TERT* gene expression (25). Furthermore, an integrative analysis of ATAC-seq and gene expression data from public repositories has identified low-grade glioma GWAS variant rs648044 as a causative SNP, which perturbed the binding affinity of the TF MAFF and thus regulated the expression of *ZBTB16* (26). In the present study, by integrating the ATAC-seq and ChIP-seq data, we discovered and validated a functional polymorphism rs13064999 in *TP63*, which associated with NSCLC susceptibility. Our results indicated that ATAC-seq data might be a helpful tool for post-GWAS strategies to localize causative SNPs.

Our function assays *in vitro* indicated that rs13064999 might regulate gene transcription by affecting HP1γ binding affinity in an allele-specific manner. HP1γ, encoded by gene chromebox protein homolog 3 (*CBX3*), belongs to a class of nonhistone chromosomal proteins involved in heterochromatin formation (27). Previous evidence have demonstrated that HP1γ plays critical roles in DNA repair (28), RNA splicing (29), and transcriptional regulation (30). In the recent year, HP1γ has been found to promote the progression of multiple cancers, such as tongue squamous cell carcinoma (31), lung cancer (32), and pancreatic cancer (33). In tumorigenesis, HP1γ was thought to regulate gene transcription through recognizing and binding to H3K9me2/3, resulting in signaling pathway alterations and the changes in tumor cellular processes. For example, HP1γ promoted proliferation and survival of prostate cancer cell by repressing miR-451a expression (34). Additionally, HP1γ was reported to promote colon cancer cell proliferation by suppressing *p21* expression (35). Nevertheless, a study published by Chen et al. found that HP1γ promoted cell cycle transition of pancreatic adenocarcinoma cell *via* increasing the expression of *CDK1* and *PCNA* (36). It has been also reported that HP1γ/HP1α cooperated with intracellular MMP3 to induce the transcription of *HSP70B* gene (37). Thus, HP1γ may play dual roles in transcriptional regulation with both activator and suppressor properties. In our study, EMSA experiments revealed the specific affinity of HP1γ to the rs13064999 risk allele A, and higher luciferase activity was observed in this allele in the reporter gene assays. These results suggested that HP1γ is likely to act as a transcription activator to facilitate gene transcription by preferentially binding to the risk allele A of rs13064999. Interestingly, the reporter assays revealed that luciferase activity of the reconstructed plasmid containing rs13064999 A allele in adenocarcinoma cells (A549) was lower than the pGL3-Promoter vector, while the one in squamous cell line (SK-MES-1) was higher. This may be attributed to the biological heterogeneity between these two cell types. Indeed, it has been demonstrated that the regulation of transcription was subjected to a cell type-specific manner, which was determined by lineage-determining TFs (LDTFs) or signal-dependent TFs (SDTFs) (38). These cell-specific coregulators may thus jointly contribute to the diversity of transcription mode in different cell lines.

Two common variants in *TP3*, rs10937405 and rs4488809, have been identified as risk variants of lung adenocarcinoma by a previous GWAS in Japanese and Korean populations (39). The later meta-analyses confirmed associations between the above variants with the susceptibility of lung cancer, particularly in East Asians (40–42). Although the significant SNP rs13064999 identified in our study has not been reported before, it was in moderate LD (r^2^ = 0.60) with tag SNP rs4488809, which showed significant expression quantitative trait loci (eQTL) with *TP63* expression from the Genotype-Tissue Expression (GTEx) database in the lung tissue. These evidence suggested that *TP63* might be a potential target for the transcriptional regulation of rs13064999. *TP63* gene, one of p53 family member, plays an important role in epidermal development and homeostasis (43, 44). Due to two different promoters and various alternative splicing, *TP63* encodes several isoforms, which are classified into two categories, TAp63 and NH2-terminal truncated (ΔNp63). In general, TAp63 often acts as a tumor suppressor, whereas ΔNp63 may exhibit oncogenic function (45). Remarkably, ΔNp63 is the prominent isoform highly expressed in tumor tissues, particularly in squamous cell carcinomas (SCCs) (46). Large amounts of evidence have proved an oncogenic role of ΔNp63 in SCC pathogenesis (47–49). However, in the present study, stratification of NSCLC by histological subtype indicated that rs13064999 confers risk of lung adenocarcinoma but not lung SCC. The null association with SCC may be attributed to the quite small sample size of SCC, since lung SCC only accounted for approximately one quarter of all NSCLC cases in our study. On the other hand, although *TP63* amplification and expression has been reported in a small subset of lung adenocarcinoma (50–52), the biological mechanisms under the role of *TP63* remain unclear. Notably, owing to the lack of *TP63* gene expression data in individuals with different genotypes of rs13064999, whether *TP63* serves as a target gene of rs13064999 warrants further validation, and genome expression profiles in future studies are needed to define the target genes of this locus.

ATAC-seq is one of the most powerful approach for mapping accessible chromatin, and ChIP-seq provides comprehensive information of histone modifications to annotate regulatory elements in genomes. By integrating ATAC-seq with ChIP-seq data, we successfully identified a causal SNP in lung cancer GWAS signals, which indicated that these epigenetic signals might be a useful tool in excavating functional genes and variants. In addition, this study also presented a preliminary explanation for the role of rs13064999, making the association of this SNP with the risk of NSCLC biologically plausible. However, some limitations should be noted. First, the limited sample size of our case–control study might influence the power in discovering disease-associated variants with small effects. Second, only sex, age, and smoking status were adjusted in the regression model. Effects of other unknown confounding factors cannot be excluded. Finally, the functional experiments in our study only characterized the regulatory function of rs13064999. Further exploration about the downstream signaling pathways and the underling mechanisms involved in NSCLC tumorigenesis are warranted in the future.

In summary, through an integration of ATAC-seq and ChIP-seq, we identified a common variant rs13064999, which located in GWAS-identified 3q28 region, significantly associated with NSCLC risk in Chinese population. Functional experiments revealed that rs13064999 conferred NSCLC susceptibility through regulating transcription activity *via* allele-specific binding of HP1γ. Our study expanded our understanding of the etiology of lung cancer and provided a new strategy to identify causal SNPs with a multi-omic integration combining ATAC-seq and ChIP-seq.

## Data Availability Statement

The original contributions presented in the study are included in the article/**Supplementary Material**. Further inquiries can be directed to the corresponding authors.

## Ethics Statement

The studies involving human participants were reviewed and approved by Institutional Review Committee of the Tongji Hospital, Tongji Medical College, HUST. The patients/participants provided their written informed consent to participate in this study. Written informed consent was obtained from the individual(s) for the publication of any potentially identifiable images or data included in this article.

## Author Contributions

JLi and LC conceived and designed the study, supervised the study, and provided critical revision of the manuscript. JLo performed relevant experiments, carried out statistical analyses, and wrote or edited the manuscript. TL performed quality control, performed relevant experiments, and provided revision of the manuscript. YL performed quality control and analyzed and interpreted data. PY and KL collected clinical data and samples. All authors contributed to the article and approved the submitted version.

## Funding

This work was supported by the National Natural Science Foundation of China (grant numbers 81572071 and 81903394).

## Conflict of Interest

The authors declare that the research was conducted in the absence of any commercial or financial relationships that could be construed as a potential conflict of interest.

## Publisher’s Note

All claims expressed in this article are solely those of the authors and do not necessarily represent those of their affiliated organizations, or those of the publisher, the editors and the reviewers. Any product that may be evaluated in this article, or claim that may be made by its manufacturer, is not guaranteed or endorsed by the publisher.

## Supplementary Material

Supplementary Figure 1 Flow chart of candidate SNPs selection. GWAS, genome-wide association study; SNP, single-nucleotide polymorphism; LD, linkage disequilibrium; EAS, East Asian; ATAC-seq, assay for transposase-accessible chromatin using sequencing.
