# Supplementary figures and images for: A Functional Polymorphism in Accessible Chromatin Region Confers Risk of Non-Small Cell Lung Cancer in Chinese Population
Source: Front Oncol. 2021 Sep 6;11:698993. doi: 10.3389/fonc.2021.698993 (PMC8450516; doi:10.3389/fonc.2021.698993)

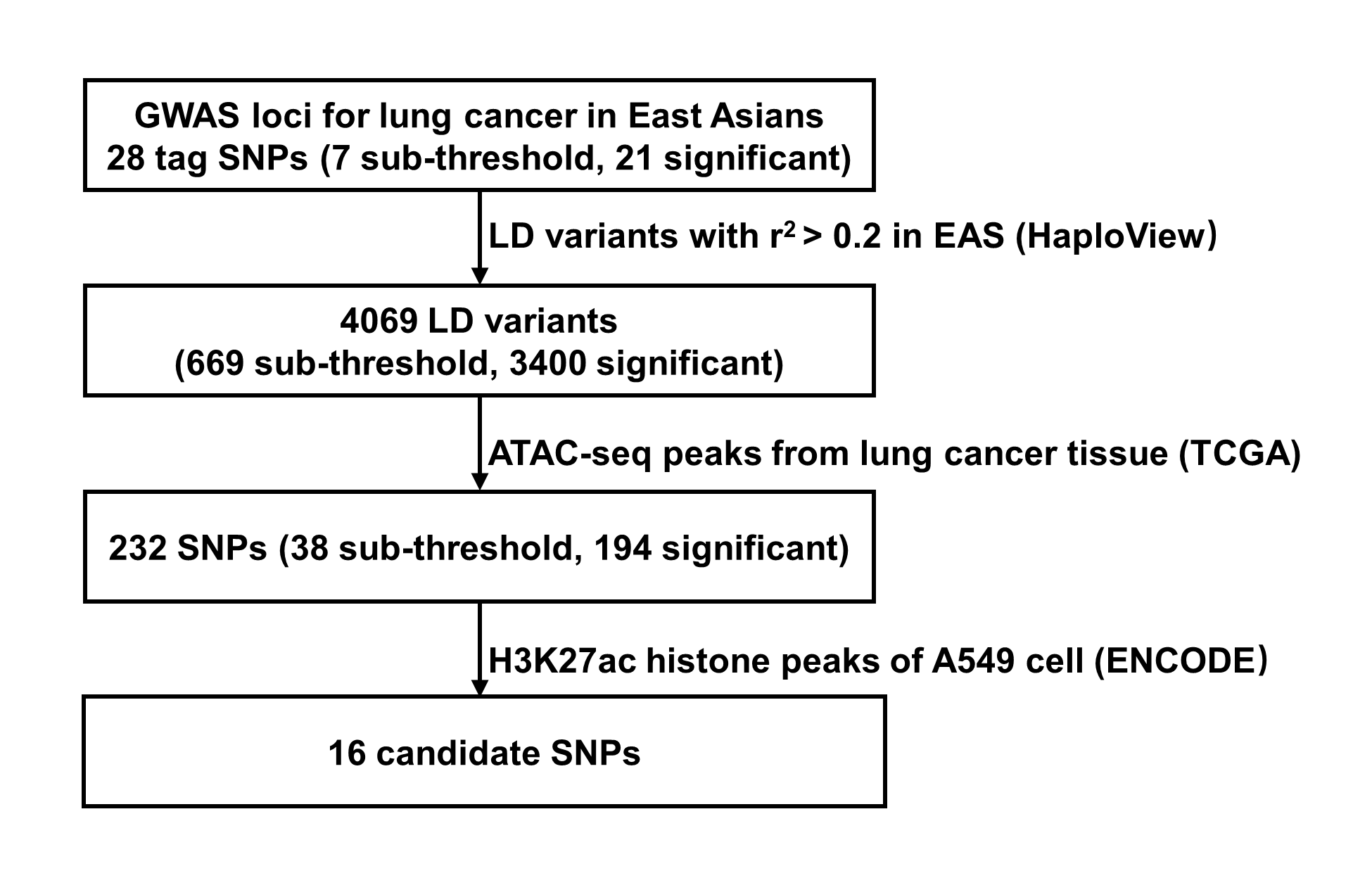

Supplement: Supplementary Figure 1 — Flow chart of candidate SNPs selection. GWAS, genome-wide association study; SNP, single-nucleotide polymorphism; LD, linkage disequilibrium; EAS, East Asian; ATAC-seq, assay for transposase-accessible chromatin using sequencing. [file Image_1.tif]
