# Supplementary material for: A Functional Polymorphism in Accessible Chromatin Region Confers Risk of Non-Small Cell Lung Cancer in Chinese Population
Source: Front Oncol. 2021 Sep 6;11:698993. doi: 10.3389/fonc.2021.698993 (PMC8450516; doi:10.3389/fonc.2021.698993)
Supplement: Supplementary file 2 [file Table_1.docx]

Table S1. Summary of 16 candidate SNPs obtained from bioinformatics analyses.

| SNP | Position | Ref | Var | MAF (ASN) | Gene | Location |
| --- | --- | --- | --- | --- | --- | --- |
| rs2290368 | chr2:151409959 | T | C | 0.47 | RIF1 | 5'-UTR |
| rs13064999 | chr3:189631712 | G | A | 0.4 | TP63 | intronic |
| rs62290287 | chr3:195102939 | G | A | 0.45 | XXYLT1 | intronic |
| rs71317943 | chr3:195102982 | A | G | 0.29 | XXYLT1 | intronic |
| rs4946258 | chr6:117482375 | C | T | 0.42 | DCBLD1 | upstream |
| rs11169971 | chr12:51977557 | T | A | 0.34 | ACVR1B | intronic |
| rs8079078 | chr17:67825050 | C | A | 0.17 | BPTF | upstream |
| rs7502307 | chr17:67993845 | C | G | 0.65 | C17orf58 | downstream |
| rs34122828 | chr17:68011163 | T | C | 0.19 | C17orf58 | upstream |
| rs151235307 | chr17:68020190 | C | T | 0.16 | KPNA2 | upstream |
| rs12752 | chr17:68035784 | T | C | 0.68 | KPNA2 | 5'-UTR |
| rs34417254 | chr17:68101288 | T | C | 0.31 | LINC00674 | upstream |
| rs12601492 | chr17:78151633 | G | T | 0.45 | C17orf99 | intronic |
| rs9906439 | chr17:78151739 | A | T | 0.27 | C17orf99 | intronic |
| rs9908003 | chr17:78168967 | G | C | 0.68 | SYNGR2 | intronic |
| rs3794742 | chr17:78169467 | G | A | 0.53 | SYNGR2 | intronic |

Abbreviations: MAF, ASN, Asian; Ref, reference allele; Var, variant allele.
